# Symptomatic Methemoglobinemia at a Relatively Low Methemoglobin Level After Aniline Exposure in a Patient With Hemoglobin D Trait

**DOI:** 10.7759/cureus.88060

**Published:** 2025-07-16

**Authors:** Omer Abdelfadiel, Yasir Abdel Latif Elbashir Ahmed, Intisar Abdulrahman, Walaa Abdelmaaboud

**Affiliations:** 1 Department of Internal Medicine, Khorfakkan Hospital, Sharjah, ARE; 2 Department of Acute Medicine, University Hospitals of North Midlands, Stoke-on-Trent, GBR; 3 Department of Intensive Care Unit, Khorfakkan Hospital, Sharjah, ARE

**Keywords:** aniline, ascorbic acid, hb d, hemoglobin d trait, methemoglobin, methemoglobinemia, methylene blue

## Abstract

Methemoglobinemia is a condition in which hemoglobin is oxidized, impairing its ability to bind and transport oxygen, leading to tissue hypoxia. We report a rare case of symptomatic methemoglobinemia in a patient with a previously undiagnosed hemoglobin D (Hb D) trait, who developed symptoms at a relatively low methemoglobin (MetHb) level following exposure to aniline. A 57-year-old male who works in a garment shop presented to the emergency department with chest pain, dyspnea, and low oxygen saturation unresponsive to high-concentration oxygen therapy. A discrepancy between arterial oxygen partial pressure (pO₂) and oxygen saturation (sO₂) raised suspicion for methemoglobinemia, which was confirmed by an elevated MetHb level of 11.3%. Hemoglobin electrophoresis revealed the presence of the Hb D trait. The patient’s recent handling of newly purchased blue jeans in a poorly ventilated stockroom implicated aniline dye as the likely cause of his methemoglobinemia. Treatment with methylene blue (MB) and ascorbic acid resulted in rapid clinical improvement and normalization of MetHb levels. This observation raises the possibility that Hb D may lower the MetHb level at which symptoms develop, a hypothesis that warrants further investigation.

## Introduction

Methemoglobin (MetHb) forms when the ferrous ion (Fe²⁺) in hemoglobin is oxidized to ferric iron (Fe³⁺), altering its structure and impairing oxygen binding and delivery, leading to tissue hypoxia [1]. Methemoglobinemia occurs when MetHb levels in erythrocytes exceed 1% [2].

Methemoglobinemia is classified as congenital or acquired. The congenital form is rare and most often results from a deficiency of cytochrome b5 reductase (Cyb5R), an enzyme essential for maintaining hemoglobin in its reduced, functional state [3]. Less commonly, it is attributable to hemoglobin M (Hb M), a variant arising from mutations in the globin genes that renders hemoglobin resistant to reduction [4].

By contrast, acquired methemoglobinemia is more common and often arises following exposure to external oxidizing agents, such as medications, toxins, and environmental pollutants [5]. Medications commonly associated with methemoglobinemia include topical anesthetics (e.g., benzocaine, lidocaine, and prilocaine), as well as dapsone and antimalarial agents, including chloroquine and primaquine [6-9]. Nitrates, chlorates, and aniline compounds are among the most frequently implicated chemical agents and environmental contaminants [10].

Clinical manifestations of methemoglobinemia closely correlate with MetHb concentrations. Levels below 10% are typically asymptomatic. Cyanosis tends to appear at concentrations of 10-20%. As levels rise to 20-30%, patients may experience symptoms such as anxiety, lightheadedness, headache, and tachycardia. Between 30 and 50%, more pronounced clinical features, including fatigue, confusion, dizziness, tachypnea, and worsening tachycardia, are observed. Levels of 50-70% can lead to severe complications, notably seizures, coma, arrhythmias, and metabolic acidosis. MetHb levels exceeding 70% are often fatal. These thresholds are based on a baseline hemoglobin of 15 g/dL; individuals with lower hemoglobin levels or underlying cardiac, pulmonary, or hematologic conditions are more likely to develop severe symptoms at lower MetHb concentrations [10]. 

Management of acquired methemoglobinemia begins with prompt identification and elimination of the precipitating factors. For symptomatic individuals, methylene blue (MB) is the first-line treatment, administered intravenously at a dose of 1-2 mg/kg of a 1% solution over five minutes. If there is no clinical improvement within 30 minutes, the dose may be repeated, not exceeding a total cumulative dose of 5.5 mg/kg. Before administering MB, it is essential to assess for G6PD deficiency, as treatment in deficient individuals carries a risk of severe hemolysis. Ascorbic acid is sometimes used as adjunctive therapy. If MB is ineffective, alternative treatments such as exchange transfusion or hyperbaric oxygen therapy may be necessary [11].

## Case presentation

A 57-year-old Indian male working in a garment shop presented to the ED with a two-day history of left-sided chest pain that worsened with deep breathing and was accompanied by mild dyspnea. He denied fever, cough, hemoptysis, or other systemic symptoms. He had no significant past medical history, was not on chronic medications, and reported no recent contact with sick individuals.

On examination, the patient was conscious, oriented, and alert, with no signs of respiratory distress. Vital signs were within normal limits, except for an oxygen saturation of 88-90%, which remained unchanged despite oxygen therapy via non-rebreather mask (NRM) or high-flow nasal cannula (HFNC). General physical examination revealed no pallor, edema, or clinically apparent cyanosis. Chest auscultation demonstrated normal breath sounds without wheezes or crackles. Cardiac examination was normal, with no murmurs, gallops, or rubs. The remainder of the systemic examination was unremarkable.

Initial laboratory investigations, including CBC, renal and liver function tests (RFTs and LFTs), D-dimer, and inflammatory and cardiac markers, were within reference ranges (Table 1).

**Table 1:** Laboratory investigations. g/dL: grams per deciliter; ×10³/mcL: thousands per microliter; mmol/L: millimoles per liter; g/L: grams per liter; IU/L: international units per liter; µmol/L: micromoles per liter; ng/L: nanograms per liter; mg/dL: milligrams per deciliter; mcg/L or µg/L: micrograms per liter; mg/L: milligrams per liter; mmHg: millimeters of mercury; mEq/L: milliequivalents per liter; CO₂: carbon dioxide; pCO₂: partial pressure of carbon dioxide; pO₂: partial pressure of oxygen; HCO₃⁻: bicarbonate; G6PD: glucose-6-phosphate dehydrogenase; QI: quantitative interpretation; PCR: polymerase chain reaction; HFNC: high-flow nasal cannula; MetHb: methemoglobin; Hb: hemoglobin; Hb A: adult hemoglobin; Hb A2: minor adult hemoglobin; Hb D: hemoglobin D variant; Hb F: fetal hemoglobin; Hb AD: heterozygous state of hemoglobin A and D (Hb D trait).

Test	Patient’s Result	Reference Range
CBC		
Hemoglobin (g/dL)	13.6	13.0-17.0
White Blood Cell Count (×10³/mcL)	5.7	4.0-10.0
Platelet Count (×10³/mcL)	305	150-450
Chemistry		
Sodium (mmol/L)	136	136-145
Potassium (mmol/L)	3.59	3.5-5.10
Chloride (mmol/L)	101	98-107
CO₂ (mmol/L)	26	21-32
Glucose (mmol/L)	7	3.9-6.1
Creatinine (µmol/L)	85.11	62-115
Total Protein (g/L)	70.98	64.0-82.0
Albumin (g/L)	38.1	34.0-50.0
Alanine Transaminase (ALT, IU/L)	22.73	30.0-65.0
Aspartate Transaminase (AST, IU/L)	11	15.0-37.0
Total Bilirubin (µmol/L)	5.3	3.0-17.0
Alkaline Phosphatase (IU/L)	62.4	46.0-116.0
Troponin (ng/L)	4.984	0.0-60.0
D-Dimer (mg/dL)	0.19	0.0-0.5
Ferritin (mcg/L)	130	26.0-388.0
Lactate Dehydrogenase (LDH, IU/L)	145	82.0-227.0
Procalcitonin (µg/L)	0.01	≤0.10
C-Reactive Protein (CRP, mg/L)	0.9	0.0-3.0
Lactate (mmol/L)	2.2	0.5-2.0
G6PD (QI)	Normal	-
COVID-19 PCR	Not detected	-
Venous Blood Gas at Presentation		
pH	7.35	7.34-7.45
pCO₂ (mmHg)	51	41-51
pO₂ (mmHg)	36	30-50
HCO₃⁻ (mmol/L)	27	23-28
Arterial Blood Gas on HFNC		
pH	7.415	7.35-7.45
pCO₂ (mmHg)	35.9	35-45
pO₂ (mmHg)	257.8	80-100
HCO₃⁻ (mmol/L)	22.5	23-28
Base Excess (mEq/L)	-1.5	-2.0 to +2.0
Methemoglobin (MetHb, %)	11.3	0.0-2.0
Hemoglobin Electrophoresis		
Hb A (%)	50.2	95-98
Hb A2 (%)	1.5	2.0-3.0
Hb D (%)	37.1	0
Hb F (%)	0.3	0.8-2.0
Interpretation	Hb D Trait (Hb AD)	-

**Figure 1:**
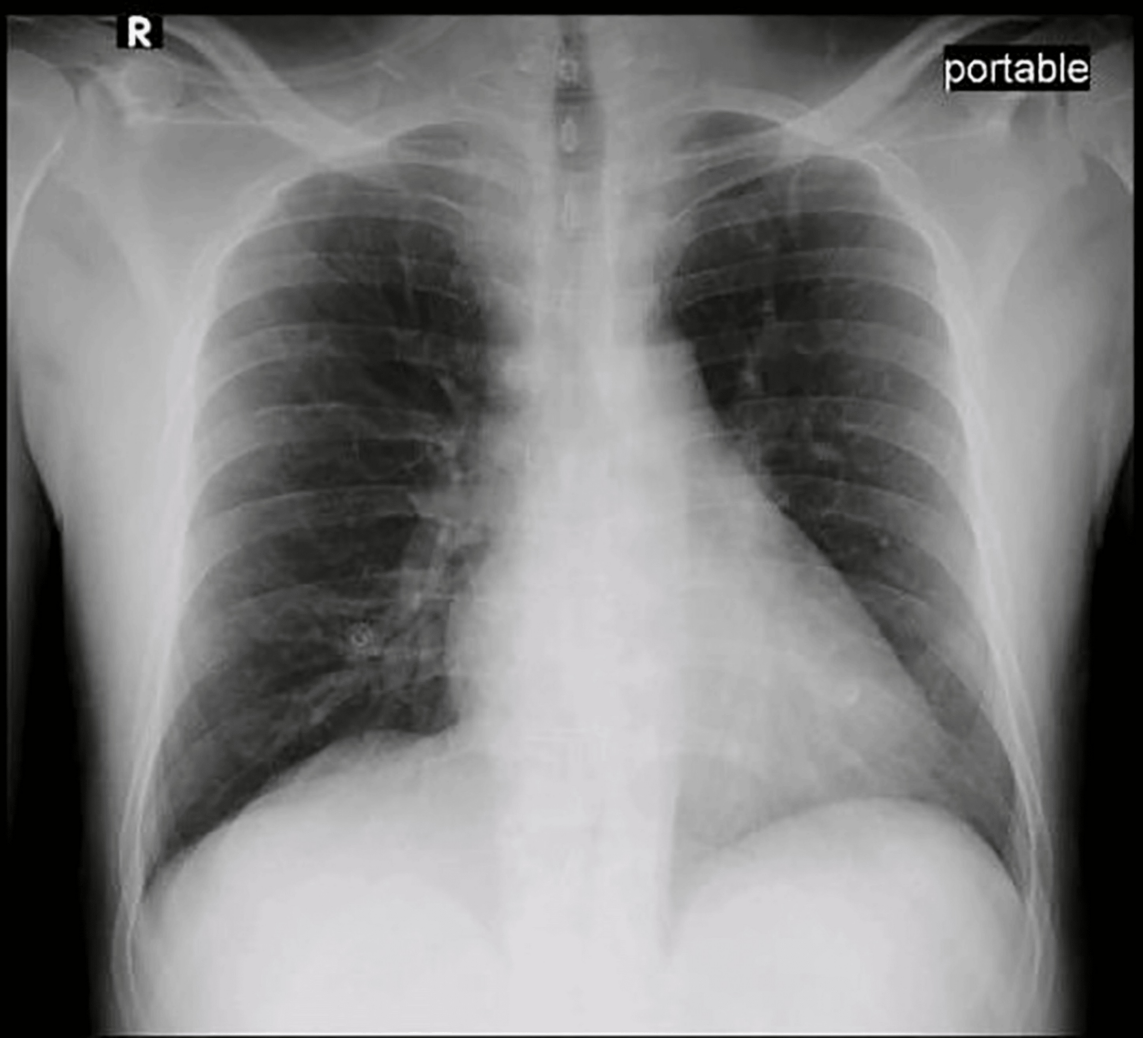
Chest X-ray.

**Figure 2:**
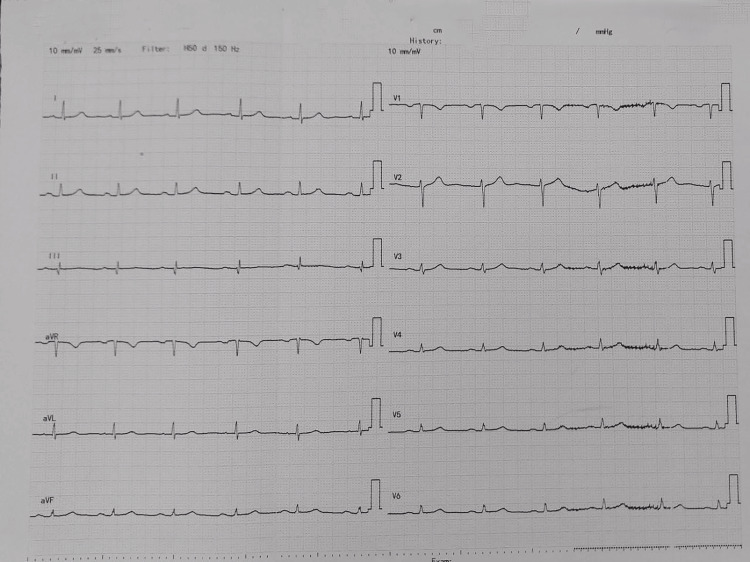
ECG of the patient.

**Figure 3:**
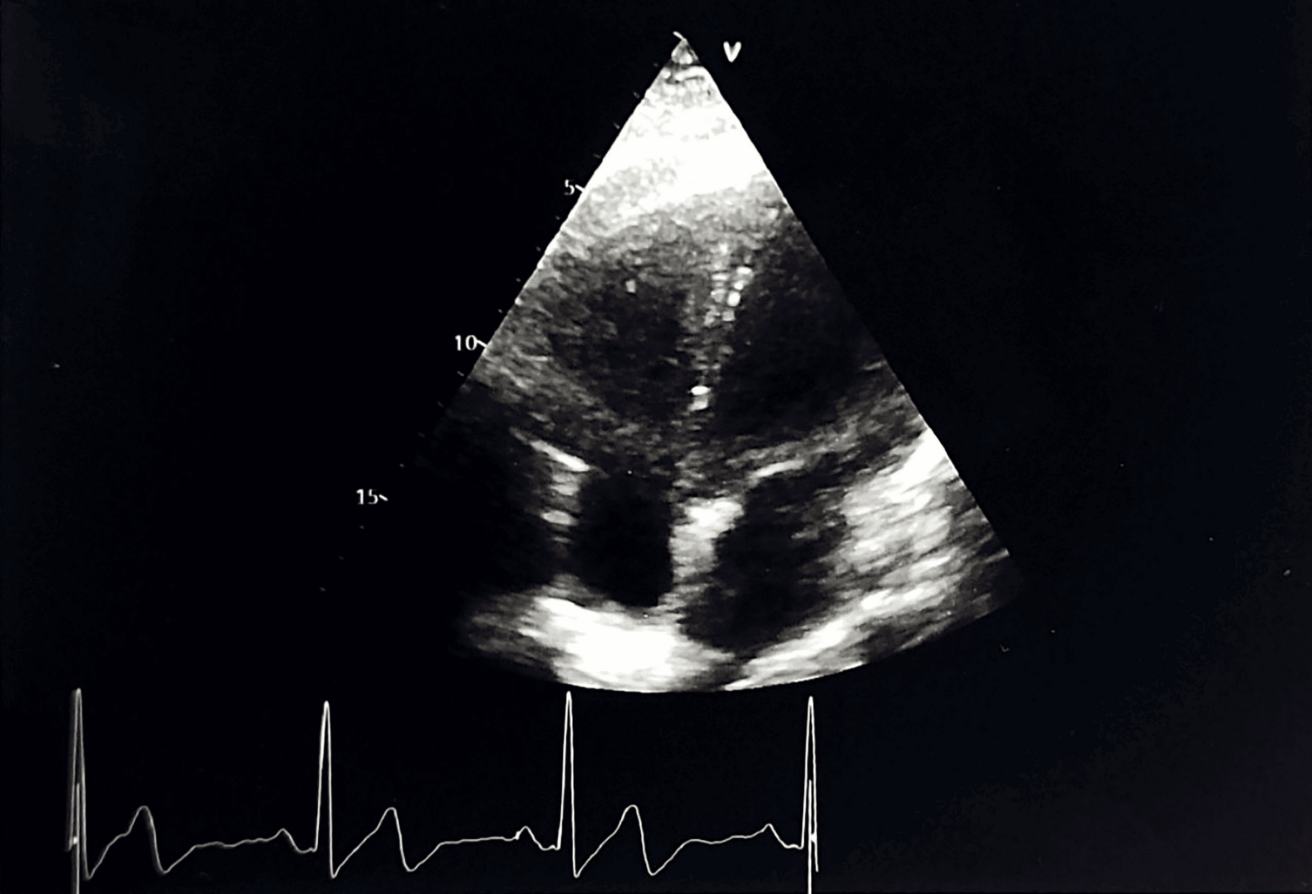
Transthoracic echocardiogram (apical four-chamber view).

Chest X-ray (Figure 1), ECG (Figure 2), and transthoracic echocardiography (Figure 3) showed no abnormalities.

Arterial blood gas (ABG) analysis on HFNC (FiO₂ 60% and FR 60 L/min) showed a pH of 7.41, pCO₂ of 35.9 mmHg, pO₂ of 257.8 mmHg, and HCO₃⁻ of 22.5 mmol/L. The marked discrepancy between elevated pO₂ and low sO₂ raised suspicion of methemoglobinemia, prompting assessment of MetHb level, which was found to be elevated at 11.3%. Hemoglobin electrophoresis subsequently revealed the presence of hemoglobin D (Hb D) trait (Table 1).

Upon further questioning, the patient reported that his symptoms began shortly after handling newly purchased blue jeans in a poorly ventilated stockroom at his workplace. This exposure, combined with the confined setting, strongly implicates aniline dye as the likely trigger for his methemoglobinemia.

Following the diagnosis, the patient was treated with a single dose of MB (70 mg) and ascorbic acid, which led to rapid clinical improvement. His oxygen saturation normalized to 98-100% on room air, MetHb levels decreased to 1.0%, and his symptoms fully resolved. He remained clinically stable and was discharged after a three-day hospital stay.

## Discussion

Methemoglobinemia is a rare but clinically significant condition that arises from the oxidation of hemoglobin iron from the ferrous (Fe²⁺) to the ferric (Fe³⁺) state, which renders hemoglobin unable to effectively bind or deliver oxygen, resulting in functional anemia and tissue hypoxia. The severity of clinical manifestations tends to correlate with MetHb levels, with cyanosis frequently observed at concentrations between 10-20%, and more pronounced symptoms occurring when levels exceed 20%. However, in our case, the patient exhibited symptoms at a MetHb level of 11.3%, which is below the expected symptomatic threshold.

Aniline is an aromatic amine with a broad range of industrial applications, functioning as an intermediate in the synthesis of isocyanates, rubber-processing chemicals, dyes, pigments, agricultural agents, and pharmaceuticals [12]. In the dye industry, it plays a key role in the production of indigo, the characteristic blue dye used in the manufacturing of denim for blue jeans [13]. Occupational exposure to aniline is a well-documented risk factor for methemoglobinemia, particularly in industrial settings such as dye and chemical manufacturing. However, indirect exposure in non-industrial environments, such as handling newly manufactured denim garments in retail or garment shops, may also pose a risk due to residual aniline-based dyes, as demonstrated by the present case.

The pathogenesis of aniline-induced methemoglobinemia involves its hepatic metabolism to reactive intermediates, most notably phenylhydroxylamine, which directly oxidizes hemoglobin to methemoglobin [14]. Harrison JH Jr. and Jollow DJ demonstrated that phenylhydroxylamine is the only aniline metabolite to accumulate in the blood at concentrations sufficient to induce MetHb formation in rats, underscoring its central role in mediating toxicity [14]. Consistent with this, Jenkins FP et al. confirmed phenylhydroxylamine as the key mediator of toxicity in humans, observed that its oxidative activity is enhanced by glucose, and noted that aniline is more toxic in humans than in rats, with a no-effect dose in adults estimated at approximately 15 mg [15].

Aniline can be systemically absorbed through ingestion, inhalation, and dermal contact, and several cases of methemoglobinemia have been reported following exposure via each of these routes. Kearney TE et al. described a case of severe methemoglobinemia caused by accidental ingestion of a gasoline octane booster composed entirely of aniline, which had been mistaken for a soft drink [16]. Ravi KY et al. documented two cases of methemoglobinemia attributed to inhalation of aniline fumes in pharmaceutical manufacturing settings [17]. In another report, Lee CH et al. presented a case of methemoglobinemia due to extensive facial and upper body dermal contact with aniline, with a MetHb level reaching 46.8% [18]. Additionally, Noronha N et al. reported a case of methemoglobinemia in a 4-year-old boy who developed symptoms after wearing newly painted shoes, presumed to contain aniline-based dye [19].

In our case, the most plausible routes of aniline exposure were inhalation and dermal absorption, as the patient developed symptoms shortly after handling newly purchased blue jeans in a poorly ventilated storage area. Although the workplace environment was not formally assessed, the absence of other known chemical or pharmacological exposures strongly suggests that the aniline dye in the denim was the precipitating factor. Given these findings, exposure to consumer products, such as clothing treated with aniline-based dyes, should be considered when evaluating cases of unexplained methemoglobinemia, particularly when conventional chemical or pharmacologic exposures are not apparent.

Notably, our patient experienced chest pain and mild dyspnea at a MetHb level of 11.3%,a concentration generally associated with cyanosis rather than other clinical manifestations. Gafaar B et al. documented a similar case involving severe chest pain at a higher MetHb level [20]. This suggests that, while rare, chest pain may occur in select individuals with methemoglobinemia and should be recognized as a potential, albeit uncommon, clinical feature.

A noteworthy finding in this case is the co-existence of the Hb D trait, a hemoglobin variant first described by Itano in 1951 [21]. Hb D, also known as Hb D-Punjab or Hb D-Los Angeles, results from a substitution of glutamic acid with glutamine at codon 121 of the β-globin gene [β121(GH4) Glu→Gln] [22]. It is among the most common hemoglobin variants globally, with a higher prevalence in the Punjab region of India, and ranks as the third most common variant in Brazil [23]. Hb D is inherited in homozygous (Hb DD), heterozygous (Hb AD), or compound heterozygous forms with other hemoglobinopathies [24]. In individuals with the Hb D trait, hemoglobin electrophoresis typically reveals Hb D levels ranging from 25% to 44%, while homozygous individuals exhibit markedly higher levels, between 87% and 95% [25]. In our patient, the Hb D level was 34.4%, consistent with a heterozygous state.

Hb D disease (Hb DD) is rare and typically presents with mild hemolytic anemia and splenomegaly. Individuals with the Hb D trait are usually asymptomatic; however, coinheritance with Hb S or β-thalassemia can lead to sickle cell disease or chronic hemolytic anemia [26].

The co-inheritance of the Hb D trait in our patient raises the possibility that this hemoglobin variant may contribute to symptom development at lower MetHb levels than generally expected. While the clinical significance of this observation remains uncertain, it warrants further investigation into a potential link between Hb D and the expression of methemoglobinemia. To our knowledge, this is the first reported case of symptomatic methemoglobinemia in an individual with the Hb D trait, highlighting an area for future research.

## Conclusions

This case highlights the importance of maintaining a high index of suspicion for methemoglobinemia in patients presenting with unexplained hypoxia or a saturation gap, particularly following exposure to aniline, even from consumer products such as dyed garments. It also raises the possibility that hemoglobin variants, Hb D in particular, may lower the threshold of MetHb levels required for symptom development, a hypothesis that warrants further investigation.
